# PINet 1.0: A pathway network-based evaluation of drug combinations for the management of specific diseases

**DOI:** 10.3389/fmolb.2022.971768

**Published:** 2022-10-18

**Authors:** Yongkai Hong, Dantian Chen, Yaqing Jin, Mian Zu, Yin Zhang

**Affiliations:** Institute of Health Service and Transfusion Medicine, Academy of Military Medical Sciences, Beijing, China

**Keywords:** pathway, gene, drug combination, network pharmacology, random walk with restart

## Abstract

Drug combinations can increase the therapeutic effect by reducing the level of toxicity and the occurrence of drug resistance. Therefore, several drug combinations are often used in the management of complex diseases. However, due to the exponential growth in drug development, it would be impractical to evaluate all combinations through experiments. In view of this, we developed Pathway Interaction Network (PINet) biological model to estimate the optimal drug combinations for various diseases. The random walk with restart (RWR) algorithm was used to capture the “disease state” and “drug state,” while PINet was used to evaluate the optimal drug combinations and the high-order drug combination^1^. The model achieved a mean area under the curve of a receiver operating characteristic curve of 0.885. In addition, for some diseases, PINet predicted the optimal drug combination. For example, in the case of acute myeloid leukemia, PINet correctly predicted midostaurin and gemtuzumab as effective drug combinations, as demonstrated by the results of a Phase-I clinical trial. Moreover, PINet also correctly predicted the potential drug combinations for diseases that lacked a training dataset that could not be predicted using standard machine learning models.

## 1 Introduction

Compared with the “one disease, one gene” drug paradigm, drug combinations can more effectively cope with multifactorial diseases such as infections, cardiovascular diseases, and tumors (Bayat Mokhtari et al., 2017) (Huffman et al., 2017). Drug combinations can also delay the development of drug resistance and are often used in the treatment of acquired immunodeficiency syndrome (AIDS) and multi-drug resistant bacteria (Liu et al., 2021) (Cihlar and Fordyce, 2016). Network or multi-pharmacology involves the combinations of several drugs used for different targets to create a synergistic effect that can perturb the biological networks and thus increase the clinical benefits (Jia et al., 2009).

The development of optimal drug combinations typically involves three stages: the intuition phase, the clinical trials phase, and the biological data mining phase. However, since the development of the current drug combination is based on the researchers’ intuition and expertise, the process is often inefficient. As a result, it is now gradually being replaced by the high-throughput screening method (Shinn et al., 2019). Nevertheless, as the number of approved drugs increases, the number of drug combinations requiring high-throughput screening verification has increased exponentially, eventually leading to a significant prolongation of the verification process and research costs. Machine learning and deep learning, which can mine the correlation between massive amounts of biological data, are increasingly being used in the discovery of effective drug combinations (Shi et al., 2018) (Li et al., 2020) (Kim et al., 2021) (Zagidullin et al., 2021). Since machine learning depends on training datasets, it is mostly used for tumors. However, for diseases that lack training datasets, the model is difficult to optimize because it is not possible to fit the parameters into the model. In addition, the results provided by the machine learning algorithms are often difficult to explain, and therefore clinicians find it difficult to apply the machine learning solution in clinical practice. An alternative approach to the data-driven machine learning method is to use theory-driven methods based on the knowledge of biological systems and networks (Wang et al., 2021) (Jafari et al., 2022). Compared with data-driven methods, theory-driven methods are more explanatory, and their performance is not affected by the quality of the training dataset. The limitation of theory-driven methods is that they rely on the accurate generation of a theoretical hypothesis.

(Yang et al., 2008) define two network biological states: the disease and normal states. According to (Yang et al., 2008), the transition from the disease state to the normal state is achieved through the perturbation of specific target combinations within the arachidonic acid network (a kind of inflammation-related network). This approach has several limitations. First of all, there is a lack of uniform standards to define the disease and normal states. Therefore, the definition of these states often requires the subjective input of expert professionals. In addition, not all disease targets have corresponding drugs available, and more than one pathway may be involved in the development of a specific disease (Geva-Zatorsky et al., 2010). found that the protein responses to drug combinations can be accurately described by a linear superposition (weighted sum) of each protein’s response to each specific individual drug. Based on this finding (Lee et al., 2012), made use of gene set enrichment analysis to convert the gene expression profile of specific cancers (non-small cell lung cancer and triple-negative breast cancer) into related signaling pathways. The data about the linear drug superposition combinations was combined with the disease pathways data to obtain the optimal drug combination. Through this method (Lee et al., 2012), found two combination drug pairs with a synergistic effect on lung cancer cells. However, this method still has a number of shortcomings since it ignores the relationship between pathways. Moreover, the theory of linear superposition does not fit all kinds of protein. Because drugs acting on the same pathway through different targets or drugs regulating a relatively small number of highly-connected pathways are more likely to produce synergistic effects (Chen et al., 2016), proposed a “pathway to pathway interaction” network model to predict the therapeutic effect of synergistic drug combinations. This model resulted in an area under the curve (AUC) of a receiver operating characteristic curve of 0.75. The method proposed by (Chen et al., 2016) still has some shortcomings. This method ignores the disease condition, and only the pathway associations of gene overlap are retained, while the pathway associations of protein interactions and function associations are discarded. In addition, the drug combinations are evaluated based on the shortest path without considering the global topology features^2^. Therefore (Cheng et al., 2019) quantifyied the network-based relationship between drug targets and the diseased human protein to protein interaction. Although this method revealed the existence of six distinct potiential drug combinations, only one of these six drug combinations correlated with therapeutic effects. Eventually, a beneficial therapeutic effect was noted when the drug targets hit the same disease module located in separate neighborhoods. Still, the application of this model is limited as it ignores the pathway information and uses the shortest path to evaluate the optimal drug combinations without considering the global topology features.

In view of this, we constructed a Pathway Interaction Network (PINet) model to overcome the limitations of the models described in previous studies (Table 1). This new model abstracts the human body as a two-layer network containing gene and pathway information and describes the influence of a disease or drug on the human as a probability distribution in the network, which is called “disease state” and “drug state.” In addition, it predicts the optimal drug combinations by combining “disease state” and “drug state”.

**TABLE 1:** Optimization of previous research.

Inadequacies of predecessors	Improvement measures
Ignore global topology features Chen et al. (2016), Cheng et al. (2019)	Analyzing networks using RWR
Ignore pathway information Yang et al. (2008), Cheng et al. (2019)	Building a two-layer heterogeneous network
It is difficult for users to select indicators Yang et al. (2008)	Redefine disease states without user selection
Only applicable to 1 or 2 diseases Yang et al. (2008), Lee et al. (2012) Chen et al. (2016), Cheng et al. (2019)	The new model incorporated multiple diseases and the sensitivity of the specific disease was validated

The main advantage of the PINet model over the other models is that it can evaluate 5-drug combinations, while most models can only evaluate 2-drug combinations. In addition, PINet is also sensitive to various diseases.

## 2 Dataset

PINet is composed of four types of entities and eight types of relationships^3^: The four types of entities include pathways, genes, drugs and diseases, while the eight types of interactions include pathway to pathway, pathway to gene, gene to gene, drug to gene, disease to gene, disease to pathway, drug to disease and drug to pathway. Except for drug to disease, other data come from databases (Table 2). The specific data cleaning and processing methods are described in the Supplementary Material S1.1; Supplementary Material S1.2.

**TABLE 2:** Data source.

Data	Number	Source
Pathway	345	KEGG
Gene	18,532	STRING, KEGG, HVIDB, DrugBank, BindingDB, CTD
Drug	6,259	DrugBank, BindingDB
Disease	8	CTD, KEGG
Pathway-pathway	1,659	KEGG
Pathway-gene	34,426	KEGG
Gene-gene	5,680,317	STRING, HVIDB
Drug-gene	39,805	DrugBank, BindingDB
Drug-pathway	57,067	KEGG enrichment analysis
Disease-gene	683	CTD
Disease-pathway	10	KEGG
Drug-disease	257	Clinical guidelines (Table 3)

**TABLE 3:** Disease-specific drug combinations.

Disease	Drug combinations	Clinical guidelines	References
acquired immunodeficiency syndrome (AIDS)	13	Office of AIDS Research Advisory Council (OARAC)	https://clinicalinfo.hiv.gov/en/guidelines/adult-and-adolescent-arv
inflammatory bowel disease (IBD)	34	The American Gastroenterological Association (AGA)	Terdiman et al. (2013), Ko et al. (2019), Feuerstein et al. (2020), Feuerstein et al. (2021)
Diabetes^*^	32	the American Diabetes Association (ADA)	American Diabetes (2021)
Atherosclerosis	63	the American College of Cardiology (ACC)	Grundy et al. (2019), Kumbhani et al. (2021), Virani et al. (2021)
acute myeloid leukemia (AML)	25	The National Comprehensive Cancer Network (NCCN)	https://www.nccn.org/guidelines/category_1
Breast cancer	60		
Non-small cell lung cancer (NSCLC)	30		

Diabetes including type 1 and type 2 diabetes.

Databases include KEGG (Kanehisa et al., 2021), STRING (Szklarczyk et al., 2021), DrugBank (Wishart et al., 2018), BindingDB (Gilson et al., 2016), CTD (Davis et al., 2021) and HVIDB (Yang et al., 2021).

## 3 Methods

### 3.1 The theoretical basis of the model

The theoretical basis of the model was built based on the findings of four studies (Yang et al., 2008). Showed that perturbing the targets can shift the disease state to the normal state. Based on this study, we introduce the probability distribution of different drugs or diseases in the network as drug states or disease states and the higher degree of overlap between the drug state and the disease state, the better the efficacy of the drug. Chen et al. (2016). showed that the effect of a disease or drug on the body is achieved through the manipulation of genetic pathways. Therefore, our model included information on the genes and pathways. We also made use of the work of Geva-Zatorsky et al. (2010), which simplified the drug combinations as a linear summation of drug targets. The targets of drug A within our model were denoted as (a_1_, a_2_, and a_3_), and the targets of drug B were denoted as (b_1_, b_2_). Based on the study of (Geva-Zatorsky et al., 2010), the drug state of the combination of drugs A and B was deemed to be equivalent to the drug state of the virtual drug V, of which targets are (a_1_, a_2_, a_3_, b_1_, b_2_). Finally, to narrow down the scope of potential drug combinations and reduce the computational power costs, we used the research of Cheng et al. (2019), which demonstrated that drug synergy is more likely to occur when the drugs act on different disease targets at the same time.

### 3.2 Construct network model

**FIGURE 1:**
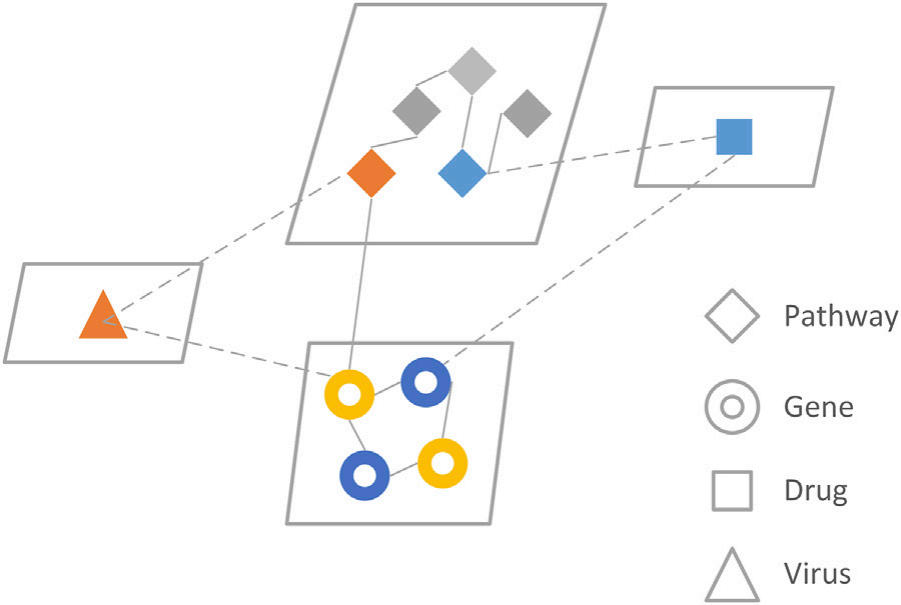
The network model of PINet. PINet model consists of four entities and seven relationships. The genes and pathways were directly related to RWR, and the drugs and viruses were integrated with the RWR algorithm through indirect connections.

PINet consists of seven networks^4^ (pathway to pathway, gene to gene, pathway to gene, drug to gene, drug to pathway, disease to pathway, disease to gene), each stored in an adjacency matrix (Figure 1). The main part of the PINet model was based on the restart random walks (RWR) algorithm built on the pathway to pathway, gene to gene, and pathway to gene networks. Further details about the model constructions are provided in the Supplementary Material S1.3 **.**

### 3.3 Capturese state

The effect of a drug or disease on the body can be represented by a vector that contains both pathway and genetic information, which is called a drug state or disease state. These two states were obtained by selecting specific initial nodes on the model to perform the RWR, and the stable probability distribution was defined as the drug or disease state. The specific state capture is described in more detail in the Supplementary Material S2 **.**

#### 3.3.1 Random walk with restart

Biological systems can be simplified into heterogeneous networks, and the RWR algorithm is widely used in the analysis of heterogeneous networks (Cho et al., 2016) (Luo et al., 2017). The RWR algorithm was developed by determining the initial probability, the transition matrix, and the stable probability distribution threshold as follows. More detail about the RWR algorithm is available in the Supplementary Material S2 **.**

##### 3.3.1.1 Determination of the initial probability

The initial nodes were composed of disease or drug-related genes and pathways. The initial probability in a specific network was composed of the initial gene to gene and pathway to pathway networks and can be calculated according to a specific node. For example, in the case of influenza, the initial gene was associated with influenza, and the initial pathway path: hsa05164 was identified from the KEGG database and was fixed to 1. On the other hand, for a drug, the original gene was considered as the drug target, the initial pathway was identified through pathway enrichment analysis, and the number of potential initial pathways was not fixed.

The initial probability of the pathway to pathway network a_0_ was formed so that equal probabilities were assigned to the initial nodes in the pathway to pathway network, and the sum of the nodes’ probabilities was equal to 1. Therefore if the probabilities of non-initial nodes are 0, then the initial probability of the gene to gene network b_0_ is the same. This relationship is summarized by the equation.

$$p_{0}=0.5\left[\begin{array}{c} a_{0}\\ b_{0} \end{array}\right]$$ (1)

Whereby *a*_*0*_ is the pathway initial probability, and *b*_*0*_ is the gene initial probability. Both *a*_*0*_ and *b*_*0*_ are vectors.

##### 3.3.1.2 Determination of the transition matrix

The transition matrix describes the transition characteristics of all nodes within the network model. There are four transfer modes in PINet: pathway to pathway, pathway to gene, gene to gene, and gene to the pathway. Each transfer mode requires a transition matrix. The description of the PINet transition node requires a large transition matrix M composed of four small transition matrices M_i_.

The (t) th probability distribution was obtained by mapping the (t-1) th probability distribution through the transition matrix as follows:

$$\left(1-r\right)\left[\begin{array}{cc} M_{1} & M_{2}\\ M_{3} & M_{4} \end{array}\right]\left[\begin{array}{c} a_{t}\\ b_{t} \end{array}\right]+rp_{0}=\left[\;\begin{array}{c} a_{t+1}\\ b_{t+1} \end{array}\right]=p_{t+1}$$ (2)

Whereby *M1* is the pathway to pathway, *M2* is the gene to pathway, *M3* is the pathway to gene, and *M4* is the gene to gene. *r* is the restart probability which is generally equal to 0.5.

##### 3.3.1.3 Determination of the stable probability distribution threshold

The initial node was selected to perform the RWR. As the number of iterations increased, the probability distribution gradually became stable. When the difference in the probability distribution between the (n)th and the (n+1)th was less than the given threshold, the (n)th probability distribution was considered to be a stable probability distribution, and the threshold was generally set to 10^–10^.

#### 3.3.2 Capturing the disease state

The disease state was then captured through the identification of the initial nodes of the disease in the pathway to pathway network and, subsequently, the gene-gene network. The initial probability p_0_ of the disease was constructed, and then RWR was performed until the probability distribution became stable. The stable probability of the disease site p_n_ was then captured for the disease state.

#### 3.3.3 Capturing the drug state

The drug state was captured through the identification of the virtual drug corresponding to the drug combination. The initial probability p_0_ of the drug was determined according to the target and enrichment pathway of the virtual drug. Finally, RWR was performed until the probability distribution became stable, and the stable probability p_n_ was captured for the drug state.

### 3.4 The drug combination score

Since the drug combinations have certain indications, we evaluated the drug combinations under specific disease conditions by “drug state” and “disease state.” The same drug combinations have different scores on different disease conditions in PINet. The absolute drug score value was obtained by calculating the difference between the “drug state” and the “disease state”.

$$score=\left|S_{di}-S_{dr}\right|$$ (3)

S_di_ is the disease state, S_dr_ is the drug state.

A lower score indicates a higher likelihood of a synergistic drug combination. Further details on the calculation of the drug combination score can be found in Supplementary Material S3 **.**

### 3.5 Evaluation of pathway interaction network

During the development of PINet, it was assumed that the drug combination contained two types of information: the drug composition and the indication. Therefore two tests were performed to evaluate the sensitivity of PINet to detect disease and drug quantity. The disease sensitivity analysis assessed whether PINet can correctly identify the indications for the different drug combinations. For example, whether PINet will wrongly judge a drug designed to treat AIDS as a drug used to treat cancer. The drug quantity sensitivity analysis evaluated the ability of PINet to identify the n-drugs combination (*n* = 2, 3, 4, and 5).

#### 3.5.1 Disease sensitivity

The drug combination highlighted in the clinical guidelines of each disease was regarded as the positive gold standard treatment. The clinical indications of the drug combinations used to manage a specific disease were then modified to represent a negative example, i.e., another disease. All positive and negative examples were entered into the PINet for scoring, and the AUC under the ROC was calculated for each example. An AUC below 0.5 indicates that the PINet model was not sensitive enough to detect the disease and corresponding drug combinations, and these were therefore excluded from the model. The remaining diseases and drug combinations in the clinical guidelines were evaluated again in the next step.

#### 3.5.2 Drug quantity sensitivity

The drug combinations may include four possible options with 2, 3, 4, or 5 drugs. The sensitivity of PINet to different drug combinations was calculated as follows. First, the drug combination in the clinical guidelines was used as a positive example, and the randomly generated drug combination was used as a negative example. Subsequently, the drug status and disease status were calculated according to the drug composition and indications, respectively, as explained in Section 3.3. Then, the score for each drug combination was calculated, as explained in Section 3.4. Finally, based on the calculated score, the AUC was calculated for each drug combination.

### 3.6 Prediction of the drug combinations

#### 3.6.1 Primary potential drug combination

**FIGURE 2:**
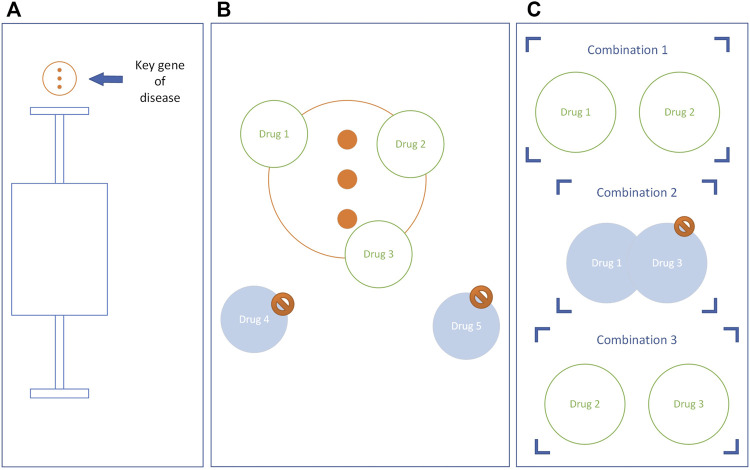
The construction of the potential drug combinations. Taking the key genes of diseases as an example, the key pathways are the same. **(A)** Genes above the upper limit are key genes. **(B)** Eliminate drugs that do not have intersections with key disease genes. **(C)** A drug combination is constructed, and if the drugs in the combination have the same target, the combination is eliminated.

Outliers of disease state are identified by Quartile, and these outliers are key genes and key pathways of the disease. The potential drugs were selected if the target of the drug had an intersection with the key gene of the disease and the enriched pathway of the drug had an intersection with the key pathway of the disease. We assumed that for N potential drugs, there are $C_{N}^{i}$ primary potential drug combinations (i is the number of drugs in the drug combination. Refer to Figures 2A–C). More detail about Quartile is available in the Supplementary Material S4 **.**

#### 3.6.2 Secondary potential drug combinations

The drug combinations with overlapping drug targets were removed from the primary potential drug combination to obtain the secondary potential drug combination (Figure 2C).

#### 3.6.3 Evaluation of the potential drug combinations

To improve the prediction accuracy of the model, we used the score corresponding to the false positive rate of 10% on the ROC of the “Drug quantity sensitivity” as the threshold. The scores of the secondary potential drug combinations were calculated, and those below the threshold were classified as synergistic drug combinations.

## 4 Results

### 4.1 Disease sensitivity

**FIGURE 3:**
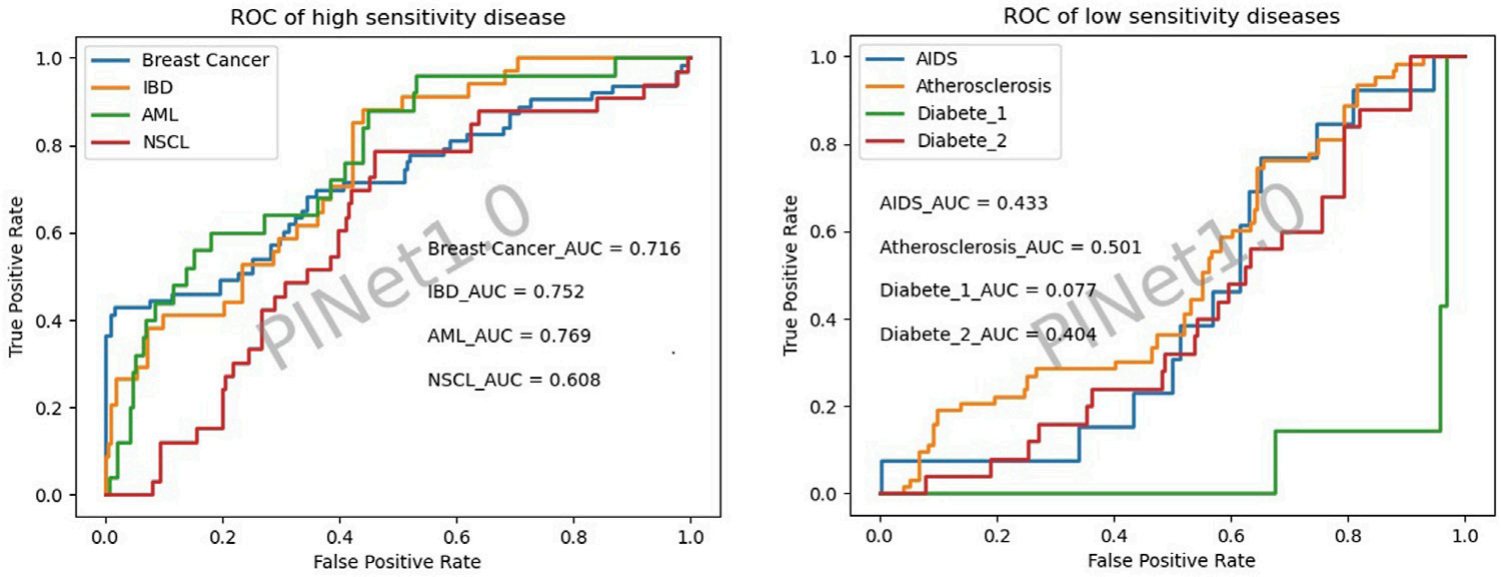
Disease sensitivity of PINet.

The PINet had a high sensitivity for NSCLC, AML, breast cancer, and IBD and low sensitivity for diabetes type 1, diabetes type 2, AIDS, and atherosclerosis (Figure 3).

### 4.2 Drug quantity sensitivity

**FIGURE 4:**
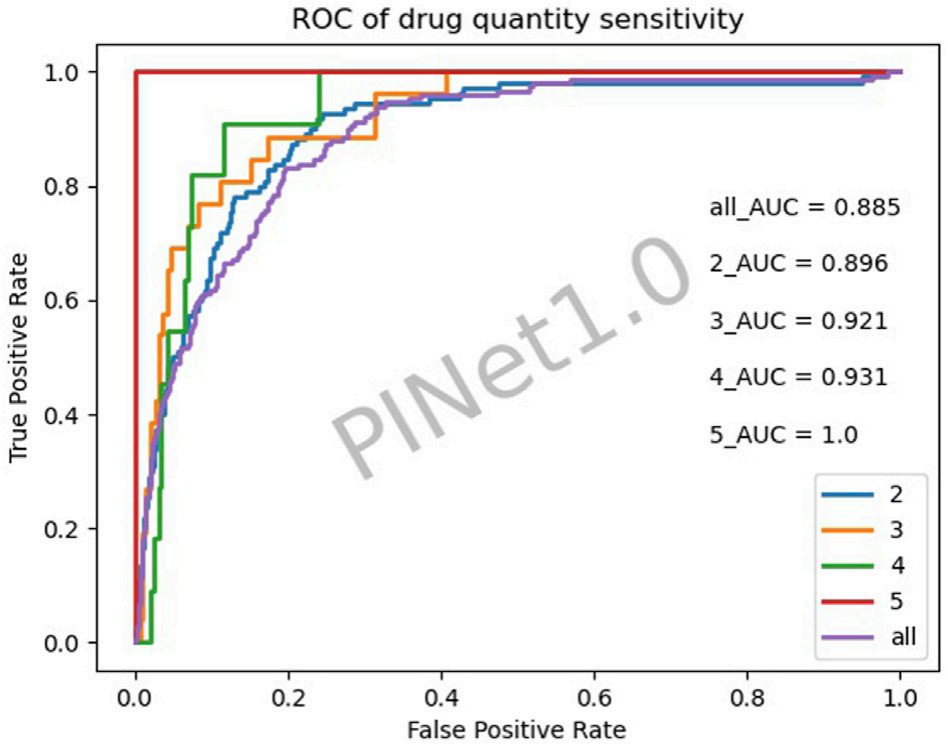
Drug quantity sensitivity of PINet.

Figure 4 illustrates the drug quantity sensitivity after excluding the diseases with a low PINet sensitivity. The sensitivity of PINet increased as the order of drug combinations increased. PINet also achieved good results in the identification of high-order drug combinations. However, since the sample was too small (2 positive cases and 58 negative cases in the fifth-order drug combination), the ROC may not be accurate.

### 4.3 Prediction accuracy

Since PINet had the highest sensitivity for predicting AML, we decided to use PINet to predict the optimal drug combinations for this disease. PINet was first used to identify the key genes and pathways of AML. Subsequently, the drugs based on these genes and pathways were identified and used to construct the primary drug combinations. This revealed a total of 26,106 possible primary drug combinations. The drug combinations with the same target were eliminated, and the remaining drug combinations (*n* = 17,713) were scored to identify the optimal drug combinations (*n* = 2,590). After excluding the unapproved drugs, 1,221 possible drug combinations were identified. The efficacy of two of the drug combinations identified by PINet has been validated in clinical trials or *in vivo* studies. Röllig et al. (2021) demonstrated the synergy between gemtuzumab ozogamicin and midostaurin in newly diagnosed AML in a phase-I clinical trial. Tian et al. (2018) found that Emricasan and Ponatinib can synergistically reduce ischemia-reperfusion injury in rat brains.

## 5 Discussion

As the development of new drugs continues to increase, there is a need to develop novel methods to identify optimal drug combinations for managing specific diseases. In this study, we proposed a novel model PINet to make it easier for clinicians to identify optimal drug combinations. When compared with other machine learning models, PINet has several advantages and limitations.

### 5.1 Advantages of pathway interaction network

#### 5.1.1 Interpretability

PINet is a theory-driven method for evaluating drug combinations based on the assumption that “drugs can correct disease states.” A low PINet score means that the drug combination is more applicable to a specific disease. This simple scoring system used in PINet is easily understood by researchers in the non-data science fields, making PINet easy to generalize.

#### 5.1.2 Non-training set dependency

Unlike machine learning, there is no need to fit all parameters in PINet, and therefore, PINet does not require a training dataset. This is crucial for drug combination prediction for some diseases that lack a training dataset.

#### 5.1.3 High-order drug combinations

Most drug combination prediction models focus on 2-drug combinations since high-order drug combinations are computationally expensive to calculate. PINet takes the same time to evaluate 2-drug combinations as higher-order drug combinations by narrowing the range of candidate drugs based on theory to maintain the computational power consumption within an acceptable range.

#### 5.1.4 Applicable to multiple diseases

A variety of diseases are already included in PINet, and the model’s effectiveness in predicting optimal drug combinations in breast cancer, IBD, AML, and NSCL has already been verified. With the advancement of disease pathway research in KEGG, the applicability of PINet will be extended to more diseases.

### 5.2 Disadvantages of pathway interaction network

#### 5.2.1 Poor sensitivity to some diseases

The sensitivity of PINet in some diseases, such as AIDS and diabetes, was found to be low in our study. A possible explanation for this could be that the effect of these diseases on genes is expressed as either an up-regulation or down-regulation gene expression. However, PINet simplifies the relationship between diseases and genes to 0 or 1, resulting in the loss of information. Furthermore, most anti-infective drugs target pathogens, and the targets of these drugs do not have corresponding genes in KEGG.

#### 5.2.2 Drug antagonism is not considered

The drug-to-target relationship was simplified to 0 or 1, and the antagonist effects of drug combinations were not considered when assessing the drug sensitivity on PINet. This means that PINet cannot distinguish between synergy and antagonism. Although we avoided competitive antagonism by narrowing down the drug candidates, this does not solve the problem on a theoretical level.

#### 5.2.3 Poor validation

The validation of PINet is not sufficient for the following reasons: Various theoretical models are suitable for different diseases, and there are certain differences in the range of drugs that can be selected, so it is difficult to make an objective comparison (Table 4). In fact, the drug combinations in PINet 1.0 are all derived from clinical guidelines, and many of these drugs lack transcriptome data and cannot be evaluated by the method of (Lee et al., 2012). There are differences between other methods (Cheng et al., 2019) (Chen et al., 2016) (Yang et al., 2008) and PINet1.0 in the indication, which makes it impossible to compare. On the other hand, due to a lack of experimental conditions, it was not possible to validate the accuracy of the PINet predictions.

**TABLE 4:** Comparison of different models.

	Yang et al. (2008)	Lee et al. (2012)	Chen et al. (2016)	Cheng et al. (2019)	PINet1.0
Indications^aa^	inflammation	NSCLC; TNBC	\	hypertension	Breast cancer; NSCLC; AML; IBD
order of drug combination^bb^	2	2	2	2	≥2
drug range^cc^	++	++	+++	+++	+++

aa: Applicable diseases of the model. bb: The number of drugs in a specific drug combination. cc: Drugs within the model. (Yang et al. (2008) only considered targets and ignored the multi-target phenomenon of drugs. Lee et al. (2012)’s drug relied on transcriptome data). NSCLC, non-small cell lung cancer; TNBC, triple-negative breast cancer; AML, acute myeloid leukemia; IBD, inflammatory bowel disease.

### 5.3 Recommendations for future practice

Several aspects can be improved on PINet to increase its prediction accuracy and applicability.

#### 5.3.1 Differentiate between synergies and indications for drug combinations

In PINet, we evaluate drug combinations by comparing disease states and drug states, considering both synergy and indications of the drug combination together. First, we found that PINet has moderate disease sensitivity but can accurately distinguish synergistic drug combinations from random drug combinations, during the evaluation of the model. In addition, the combination of drugs predicted to treat AML is suitable for ischemia-reperfusion injury, which may be related to the multi-targets phenomenon of drugs and multi-phenotypes phenomenon of diseases (Tian et al., 2018). Furthermore, synergy was identified by relying only on the shortest path in the pathway network without disease information (Chen et al., 2016). Based on the above facts, we suggest that synergy and indication should be two relatively independent attributes of a drug combination and these attributes are relatively independent and may provide a new theoretical basis for the development of a repository for the rapid identification of drug combinations. If the conjecture is correct, PINet could be used in the future to evaluate drug combinations independently of the disease state, eventually increasing the scope of application of the model. As a result, the indications can be isolated and analyzed separately in finer divisions according to the drug function (e.g., anti-inflammatory, or anti-viral) rather than the entire disease.

We plan to elucidate the synergistic effect of drug combinations through information theory. This will enable us to locate key pathways and key genes to define the indications of drug combinations and verify whether the conjecture is correct.

#### 5.3.2 Increase disease sensitivity

The relationship between diseases and genes can be optimized as −1, 0, and one to achieve differentiation of different diseases, thereby improving the disease sensitivity of PINet.

#### 5.3.3 Identify antagonism

The drug-to-target relationship can also be optimized to −1, 0, and one to simulate the antagonistic relationship between drugs. In follow-up studies, we will additionally evaluate the ability of PINet to identify antagonistic drug combinations. Chen et al., 2012, Hopkins, 2008, Hsieh et al., 2021, Zhang et al., 2021.

## Data Availability

The original contributions presented in the study are included in the article/Supplementary Material, further inquiries can be directed to the corresponding authors.

## Footnotes

2 The regulatory distance of upstream targets to downstream targets may exceed the shortest path (usually 3).

3 Relationship between drugs is predicted by the model. So it does not appear in the model. We assume that the patient has only one disease, so the relationship between diseases does not exist in the model.

4 The drug-disease relationship is the data used to evaluate the model. So it doesn't appear in the model.
