# Supplementary material for: PINet 1.0: A pathway network-based evaluation of drug combinations for the management of specific diseases
Source: Front Mol Biosci. 2022 Oct 18;9:971768. doi: 10.3389/fmolb.2022.971768 (PMC9623281; doi:10.3389/fmolb.2022.971768)
Supplement: Supplementary file 1 [file DataSheet1.PDF]

Supplementary Material

1 Data sources and processing

The PINet data can be divided into three parts. The data from each database was first obtained. A small part of the data was processed manually, and the rest was processed by the computer.

1.1 Network download

Table 1: Data source

Database	Content	Access
**KEGG**	pathway list_1	http://rest.kegg.jp/list/pathway/hsa
**DrugBank**	all drugs	https://go.drugbank.com/releases/latest
**HVIDB**	human-virus PPI data	http://zzdlab.com/hvidb/download/HVIDB_PPIs.txt
**STRING**	9606 .protein.links.v11.5 .txt.gz	https://cn.string-db.org/cgi/download?sessionId=bfmZ2o518gEC&species_text=Homo+sapiens
**CTD**	CTD_genes_diseases.tsv .gz	https://ctdbase.org/downloads/;jsessionid=AC721C7C339D7612EFBD3776AC0E8982#gd
**BindingDB**	BindingDB_All_2022m4 .tsv.zip	http://www.bindingdb.org/rwd/bind/chemsearch/marvin/SDFdownload.jsp?all_download=yes
**Guideline**		See the **Table 3** of the manuscript

1.2 Manual processing

The characteristics of the different diseases, including infectious diseases (AIDS), cardiovascular diseases (atherosclerosis), endocrine and metabolic diseases (type I diabetes, type II diabetes), tumors (breast cancer, acute myeloid leukemia, and non-small cell lung cancer) and immune disorders (inflammatory bowel disease) were processed manually.

Table 2 Manually processed data

Content	Source	Access
**Pathway List**	pathway list_1	https://github.com/hierha/PINet1.0
**Disease list**	KEGG&CTD	https://github.com/hierha/PINet1.0
**Indication of Drug Combination**	Guideline	https://github.com/hierha/PINet1.0
**Disease-Path Association**	KEGG	https://github.com/hierha/PINet1.0

1.3 Computer processing

The code for data processing can be found on GitHub: https://github.com/hierha/PINet1.0

table 3 computer-processed data

Content	Code
**Gene List of HVIDB**	1.1.1~1.1.8
**Gene List of STRING**	1.2.1~1.2.5
**Gene List of HVIDB& STRING**	1.3.1~1.3.2
**Drug Target of DrugBank**	2.1.1~2.1.4
**Drug List of DrugBank**	2.1.5
**Path-Path Association**	3.1.1~3.1.3
**Gene-Gene Association**	3.2.1~3.2.2
**Path-Gene Association**	3.3.1
**Drug Target of BindingDB**	3.4.1~3.4.3
**Drug-Gene Association**	3.4.4

**Disease-Human Gene of CTD**	3.5.1~3.5.5
**Disease-Virus Gene**	3.5.6
**Disease-Gene Association**	3.5.7
**Association Matrix (1)**	3.6.1
**Drug-Path Association**	3.6.2
**Association Matrix (2)**	3.6.3
**Drug-Disease Association**	3.7.1~3.7.2

1.3.1 Drug pathway enrichment analysis

Due to the lack of drug-to-pathway association data, we adopted a gene enrichment analysis to determine whether there was a clear association between drugs and pathways. When *p* was less than 0.05, we consider a clear association between the drug and the pathway. The formula is as follows:

$$p = 1 - \sum_{i=0}^{k-1} \frac{\binom{n}{i}\binom{M-n}{N-i}}{\binom{M}{N}}$$

M is the total number of genes in a pathway, N is the number of target genes of a drug, n is the number of target genes of the drug in the pathway, k = 0.2×n (k is an integer greater than 1)

2 Model building and state capture

A simplified version of the heterogeneous network was constructed, as shown in Figure 1. Suppose the target gene of the drug A is {1, 3, 5}, and the enrichment pathway is {1, 3}. Suppose the genes of the disease A are {1, 3, 4, 5}, and the associated pathway is {1}. For the full code, refer to: https://github.com/hierha/PINet1.0/blob/main/code/PINet/RWR.py

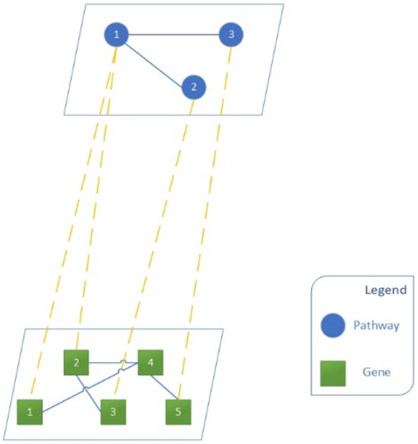

Figure 1: Simplified heterogeneous network

2.1 Initial probability

2.1.1 Identification of the initial node

For drug A, the initial node in the pathway network is {1, 3}, and the initial node in the gene network is {1, 3, 5}.

For disease A, the initial node in the pathway network is {1}, and the initial node in the gene network is {1, 3, 4, 5}.

2.1.2 Calculation of the initial probability in each network

The initial probability of the network was calculated based on the initial nodes in the network. The initial probability of the pathway network a_0 was formed such that equal probabilities are assigned to the initial nodes in the pathway to pathway network, with the sum equal to 1. The probabilities of non-initial nodes are 0. The initial probability of the gene to gene network b_0 is the same.

For drug A, the initial probability $a_0 = (1/2, 0, 1/2)^T$ in the pathway network and $b_0 = (1/3, 0, 1/3, 0, 1/3)^T$ in the gene network.

For disease A, the initial probability $a_0 = (1, 0, 0)^T$ in the pathway network and $b_0 = (1/4, 0, 1/4, 1/4, 1/4)^T$ in the gene network.

2.1.3 Calculation of the initial probability in PINet

The pathway and gene networks were deemed to be equally important and were therefore assigned the same initial probability weights in both networks. The initial probability of PINet consisted of the initial probability of the above two networks, each accounting for 50%.

For drug A, the initial probability $p_0 = (1/4, 0, 1/4, 1/6, 0, 1/6, 0, 1/6)^T$.

For disease A, the initial probability $p_0 = (1/2, 0, 0, 1/8, 0, 1/8, 1/8, 1/8)^T$.

2.2 Transition matrix

The transition matrix describes the node transition of the network in the form of probability, and the transition matrix is constant for the constructed network. The transition matrix $M_{path \to gene}$ describes the transition of nodes from the pathway network to the gene network. M is an m × n matrix, m is the number of nodes in the gene network, and n is the number of nodes in the pathway network. The first column of M describes the probability of path 1→gene 1,…, path 1→gene k, the second column describes the probability of path 2→gene 1,…, path 2→gene k, and so on.

There are four transfer modes in PINet. We first constructed 4 small transition matrices and then synthesized a large transition matrix. The construction of the large transition matrix is described in the manuscript 3.3.1.2. In this section, the construct of the small transition matrix is described in more detail. We will take pathway→pathway and pathway→gene as examples to illustrate.

Let's first look at the transfer method of pathway → pathway and the corresponding probability (Table 4). According to the above, $M_{path \to path}$ is a 3 × 3 matrix, the first column is $(0, 0.5, 0.5)^T$, the second column is $(1, 0, 0)^T$, and the third column is $(1, 0, 0)^T$ （formula-1）.

table 4 the transition of pathway→pathway

transfer method	transition probability
$path_1 \to path_2$	1/2
$path_1 \to path_3$	1/2
$path_2 \to path_1$	1
$path_3 \to path_1$	1

$$M_{path \to path} = \begin{pmatrix} 0 & 1 & 1 \\ 0.5 & 0 & 0 \\ 0.5 & 0 & 0 \end{pmatrix} \qquad \text{formula 1}$$

Then we look at the transfer method of pathway → gene and the corresponding probability (Table 5). According to the above, $M_{path \to path}$ is a 5 × 3 matrix, the first column is $(0.5, 0.5, 0, 0, 0)^T$, the second column is $(0, 0, 1, 0, 0)^T$, and the third column is $(0, 0, 0, 0, 1)^T$ （formula-2）.

table 5 the transition of pathway→gene

transfer method	transition probability
$path_1 \to gene_1$	1/2

**$path_1$→$gene_2$**	1/2
**$path_2$→$gene_3$**	1
**$path_3$→$gene_5$**	1

$$M_{\text{path}\rightarrow\text{gene}} = \begin{pmatrix} 0.5 & 0 & 0 \\ 0.5 & 0 & 0 \\ 0 & 1 & 0 \\ 0 & 0 & 0 \\ 0 & 0 & 1 \end{pmatrix} \quad \text{formula 2}$$

Finally, let's look at pathway 1. This node can move in the pathway network or run into the gene network. These two transitions are equally important in PINet, so a weight of 0.5 needs to be multiplied when constructing the final transition matrix.

2.3 Capture state

The disease index was entered, and the RWR was executed to get the disease state. The drug index was entered, and the RWR was executed to get the drug state.

3 The drug combination score

We use the Euclidean norm to consider the similarity between drug states and disease states. The smaller the score, the closer the proximity between the drug state and disease state, and the more likely the drug combination is synergistic and applicable to the disease (formula 3).

$$\|\boldsymbol{x}\| = \sum_{i}^{n} |x_i|^2 \, ; \; \boldsymbol{x} = \boldsymbol{s}_{drug} - \boldsymbol{s}_{disease}$$

$\mathbf{s}_{\text{drug}}$ is drug state, $\mathbf{s}_{\text{disease}}$ is the disease state, x_i is the element of the vector **x**.

4 Model evaluation and drug combination prediction

We first evaluated the disease sensitivity of PINet and eliminated insensitive diseases. Subsequently, we evaluated the drug combination sensitivity of PINet and finally selected highly sensitive diseases for drug combination prediction. The function of the code is shown in Table 6.

To narrow the scope of drug candidates, we first defined the normal state and then searched for the key pathways and genes of the disease by comparing the disease state with the normal state.

Disease pathways in KEGG were eliminated, and the remaining pathways were used as initial pathways. Pathogen genes were removed, and the remaining human genes were used as initial genes. The RWR was then executed to get the normal state.

4.1 Quartile

In statistics, a quartile is a type of quantile which divides the number of data points into four parts, or quarters, of more-or-less equal size. The data must be ordered from smallest to largest to compute quartiles; as such, quartiles are a form of order statistic. The three main quartiles are as follows:

- The first quartile (Q_1) is defined as the middle number between the smallest number (minimum) and the median of the data set. It is also known as the lower or 25th empirical quartile, as 25% of the data is below this point.
- The second quartile(Q_2) is the median of a data set; thus 50% of the data lies below this point.
- The third quartile (Q_3) is the middle value between the median and the highest value (maximum) of the data set. It is known as the upper or 75th empirical quartile, as 75% of the data lies below this point.

The interquartile range (IQR) is defined as the difference between the 75th and 25th percentiles of the data. so IQR = Q3 − Q1.

There is also a mathematical method to check for outliers and determining "fences", upper and lower limits from which to check for outliers.

After determining the first and third quartiles and the interquartile range as outlined above, then fences are calculated using the following formula:

$$Lower\ fence\ =\ Q_1 - 1.5 \times IQR$$

$$Upper\ fence\ =\ Q_3 + 1.5 \times IQR$$

Any data lying outside the upper fence can be considered an outlier, which are called key genes and key pathways in PINet1.0.

Table 6: The code functions

Content	Code
**normal state correlation matrix**	4.1.1~4.1.4
**disease state**	4.2.1
**key pathways and genes in disease states**	4.2.2
**building synergistic drug combinations**	5.1.1.1
**build random drug combinations**	5.1.1.2
**disease sensitivity**	5.1.2~5.1.4.3
**drug sensitivity**	5.2.1~5.2.3

**predict drug combinations**	6.1.1~6.2.1
